# Assessment of sleep quality using cardiopulmonary coupling analysis in patients with Parkinson's disease

**DOI:** 10.1002/brb3.970

**Published:** 2018-04-14

**Authors:** Lindi Chen, Chunyan Liu, Zhinan Ye, Binda Wang, Songbin He

**Affiliations:** ^1^ Department of Neurology Zhoushan Hospital Wenzhou Medical University Zhoushan China; ^2^ Department of Critical Care Medicine Huzhou Central Hospital Huzhou China; ^3^ Department of Neurology Taizhou Municipal Hospital Taizhou China

**Keywords:** cardiopulmonary coupling analysis, Parkinson's disease, pittsburgh sleep quality index, sleep quality

## Abstract

**Objectives:**

To assess the sleep quality of patients with Parkinson's disease (PD) and evaluate the effect of cardiopulmonary coupling (CPC) analysis on sleep quality and its correlation with subjective complaints in patients with PD.

**Methods:**

Our study included 42 patients with PD and 30 healthy controls. CPC analysis and the Pittsburgh Sleep Quality Index (PSQI) were used to evaluate the sleep quality of subjects.

**Results:**

High‐frequency coupling (HFC) and sleep efficiency were significantly lower in the PD than in the control group, whereas very low‐frequency coupling (VLFC) and sleep latency were significantly higher in the PD than in the control group. PSQI scores were significantly higher in the PD than in the control group (all *p *< .05). The PSQI score showed a negative correlation with the HFC ratio in the PD group (*r *= −.478, *p *= .001). Factors related to the occurrence of PD with poor sleep quality were the unified Parkinson's disease rating scale (UPDRS) score and nocturia.

**Conclusions:**

The sleep quality of patients with PD was generally decreased. CPC analysis can reflect the subjective sleep quality of patients with PD and serve as an effective sleep monitoring tool.

## 1 INTRODUCTION

Parkinson's disease (PD) is a progressive neurodegenerative disorder affecting middle‐aged populations. It is estimated that the number of patients with PD worldwide will exceed 10 million by 2030 (Dorsey et al., 2007). PD typically presents clinically with a distal resting tremor, rigidity, bradykinesia, and postural disturbances. Sleep disorders, which include insomnia, sleep apnea, excessive daytime sleepiness, and rapid eye movement sleep behavior disorder, are common in patients with PD and show a prevalence of 40–98%. A study spanning 8 years demonstrated that >50% of patients with PD reported insomnia (Gjerstad, Wentzel‐Larsen, Aarsland, & Larsen, 2007). Sleep disorders seriously affect the quality of life in patients with PD.

Currently, objective assessment of sleep physiology relies primarily on polysomnography (PSG) (Buysse, Ancoli‐Israel, Edinger, Lichstein, & Morin, 2006). PSG records the electroencephalographic (EEG) activity, extraocular eye movements, mentalis muscle tone, air flow, respiratory effort, and cardiac rhythm to determine sleep staging and aids in diagnosing different types of sleep disorders. However, the assessment of sleep quality is usually based on patient self‐reporting, interviews and psychological variables. The limitations of conventional PSG in assessing sleep quality include the following: (1) PSG may not meet the standard demographic data (Feige et al., 2008; Nofzinger et al., 2004), (2) The amplitude and morphology of EEG waves may show large individual variations, which could interfere with an accurate estimation of sleep staging and quality (Armitage, Trivedi, Hoffmann, & Rush, 1997; Berry et al., 2012), (3) The fact that the majority of sleep is characterized by second‐stage non‐rapid eye movement (NREM) sleep is a significant limitation.

Recently, the cardiopulmonary coupling (CPC) method has been used as a new method for sleep quality monitoring. This modality, which is based on the electrocardiogram (ECG) record, uses heart rate variability (HRV) and ECG R‐wave amplitude fluctuations associated with respiration to generate frequency maps of coupled autonomic–respiratory oscillations. HRV is modulated by respiration, as evidenced by shorter RR intervals during inspiration and longer RR intervals during expiration. Coupling of autonomous physiological data streams in sleep is reinforced by avoiding “wake noise” and supported by clinical observations of phase and time‐locked transients associated with fragmented sleep (e.g., increased muscle tone, heart, EEG signs of arousal, and oxygen desaturation associated with sleep apnea events). A classification of sleep based on CPC includes “stable” (high‐frequency coupling [HFC], 0.1–0.4 Hz) and “unstable” (low‐frequency coupling [LFC], 0.1–0.01 Hz), compared to standard sleep stages. Wake and rapid eye movement (REM) sleep exhibits very low‐frequency coupling (VLFC, 0.0039–0.01 Hz) (Thomas, Mietus, Peng, & Goldberger, 2005). Without a recording of muscle tone, REM sleep is not distinguishable from the wakeful state, and the detection of VLFC may reflect contributions from both states.

Sleep quality is defined in terms of subjective complaints, and diagnostic criteria notably rely on subjective measures of assessment such as scales. A variety of scales have been applied for the evaluation of sleep disorders; however, only a few scales can be used to assess sleep quality in patients with PD (Högl et al., 2010). The Pittsburgh Sleep Quality Index (PSQI) comprises 19 questions, which are used to generate 7 individual scores (subjective sleep quality, sleep latency, duration, sleep efficiency, sleep disturbances, use of sleep medications, and daytime dysfunction). Five additional questions are answered by patient's guardian but do not contribute to the final score.

In this study, we used CPC analysis and the PSQI to assess and compare the sleep quality between patients with PD and healthy subjects. We aimed to evaluate the effect of CPC analysis on sleep quality and its correlation with subjective complaints in patients with PD.

## 2 PATIENTS AND METHODS

### 2.1 Subjects

Our study included a PD group comprising 42 patients (18 men and 24 women). All patients had been registered at the Department of Neurology, Zhoushan Hospital of Zhejiang Province between October 2015 and July 2016, and written informed consent had been obtained from all patients for research purposes. In all, 40 patients were treated with Levodopa (L‐dopa) or a dopamine (DA) agonist. The daily dose of L‐dopa and DA agonist was represented as “dose‐equivalent dopa” (Krack et al., 1998; Lozano et al., 1995). PD was diagnosed based on the United Kingdom Parkinson's Disease Society Brain Bank clinical diagnostic criteria. Exclusion criteria were as follows: 1) Patients with dementia, severe anxiety, depression, or psychosis, 2) Those with other significant diseases such as cancer, heart failure, renal failure, and chronic obstructive pulmonary among others.

Additionally, we studied 30 healthy controls of similar age, dietary, and living habits as patients belonging to the PD group. No intergroup differences were noted (Table 1).

**Table 1:** Demographic and clinical data of subjects (Mean ± SD)

	PD	Control	*p*
*N*	42	30	
Sex (male/female)	18/24	13/17	.968
Age (years)	69.24 ± 6.94	68.83 ± 6.54	.803
BMI	21.28 ± 2.51	21.32 ± 2.94	.957
Education (years)	4.00 ± 3.57	3.83 ± 3.72	.867
Duration (years)	3.46 ± 2.47	–	
UPDRS	34.26 ± 17.51	–	
H‐Y	2.01 ± 0.73	–	
DA dose (mg)	271.26 ± 163.24	–	

PD, Parkinson's disease; BMI, body mass index; UPDRS, Unified Parkinson Disease Rating Scale; H‐Y, Hoehn and Yahr Scale; DA, dopamine.

### 2.2 Methods

The purpose of the study had been explained to all subjects prior to their enrollment. Detailed history taking and examination were performed in all subjects. The body mass index (BMI) and educational level were recorded. The unified Parkinson's disease rating scale III (UPDRS‐III) was assessed to identify the severity of PD, and the Hoehn and Yahr (H‐Y) classification was used to determine the stage of PD (Liu et al., 2017).

The RemLogic diagnostic software version 1.1 (Embla Systems, Inc., Thornton, CO, USA) was utilized for CPC analysis. This method collects ECG data continuously during sleep and records the HRV and ECG‐derived respiratory activity from ECG signals. The software sets the algorithm to generate a sleep spectrum. Six CPC variables are recorded: 1) HFC, 2) LFC, 3) VLFC, 4) The apnea‐hypopnea index (AHI), which determines the number of apneic events/hour, 5) Sleep latency, and 6) Sleep efficiency, which is defined as the ratio of sleep time to the total recording time.

The PSQI was administered to all subjects to obtain information regarding sleep quality and habits in patients with PD over the previous month. The PSQI summary score ranges from 0 to 21, which corresponds to increasingly impaired sleep. We defined the subjective sleep quality based on the PSQI score. A score <10 was defined as “good” sleep, and a score ≥10 was defined as “poor” sleep. We also recorded the frequency of nocturia.

The SPSS software (Inc. Chicago, IL, USA) was used for data analysis. Values were expressed as means ± standard deviations unless otherwise specified. Intergroup comparisons of primary data were performed using the Student's t test and nonparametric tests. The Chi‐square test was used to determine group distribution (Liu et al., 2017). Pearson's correlation test was used to detect the correlation between CPC variables and the PSQI scores in the PD group. The potential risk factors (including duration of PD, doses of dopa, the UPDRS score, the H‐Y classification, and frequency of nocturia) for PD in those with poor sleep quality were analyzed using quadratic unconditional logistic regression analysis.

## 3 RESULTS

### 3.1 Quantitative analysis of subjects

All enrolled subjects underwent examination. Table 1 shows the intergroup comparison of demographic and clinical data. Intergroup comparison showed no statistically significant differences in terms of gender distribution (chi‐square test: χ^2^ = 0.002, *p *= .968), age (t test: *t *= 0.25, *p *= .803), BMI (t test: *t *= −0.05, *p *= .957), educational level (t test: *t *= 0.192, *p *= .867).

### 3.2 Intergroup comparison of CPC variables

**Figure 1:**
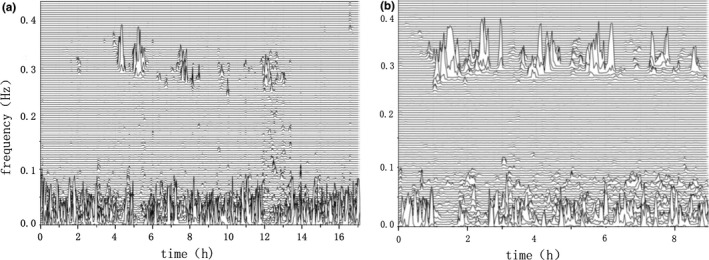
Sleep spectrums of subjects

**Figure 2:**
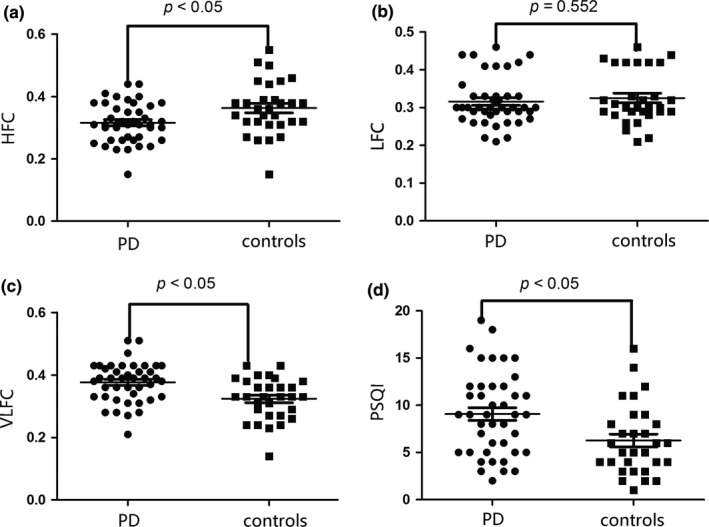
Comparisons of CPC variables and the PQSI scores

Figure 1 shows the sleep spectrums of a patient with PD versus those of a healthy participant. Intergroup comparison showed that HFC (t test: *t *= −2.737, *p *= .008) and sleep efficiency (nonparametric test: *p *= .022) were significantly lower in the PD than in the control group, whereas the VLFC (t test: *t *= 3.376, *p *= .001) and sleep latency (t test: *t *= −0.598, *p *= .552) were significantly higher in the PD than in the control group. Other CPC variables such as the LFC (t test: *t *= −0.598, *p *= .552) and AHI (nonparametric test, *p *= .125) did not show statistically significant differences (Table 2, Figure 2).

**Table 2:** Intergroup comparison of CPC variables and the PSQI scores

	PD	Control	*p*
HFC, %	31.60 ± 6.35	36.37 ± 8.48	.008*
LFC, %	31.62 ± 6.36	32.56 ± 6.94	.552
VLFC, %	37.62 ± 6.35	32.37 ± 6.72	.001**
AHI	7.48 ± 8.09	3.92 ± 5.22	.125
Sleep latency (m)	36.93 ± 19.17	27.43 ± 14.85	.026*
Sleep efficiency, %	72.81 ± 7.48	76.06 ± 4.82	.022*
PSQI	9.07 ± 4.38	6.27 ± 3.71	.006**

PD, Parkinson's disease; AHI, apnea‐hypopnea index; PSQI, the Pittsburgh sleep quality index.

*p < .05; **p < .01.

### 3.3 Intergroup comparison of the PSQI

Intergroup comparison showed that the PSQI scores were significantly higher in the PD than in the control group (t test: *t *= 2.852, *p *= .006) (Table 2, Figure 2).

### 3.4 Correlation between the PSQI score and CPC variables in the PD group

**Figure 3:**
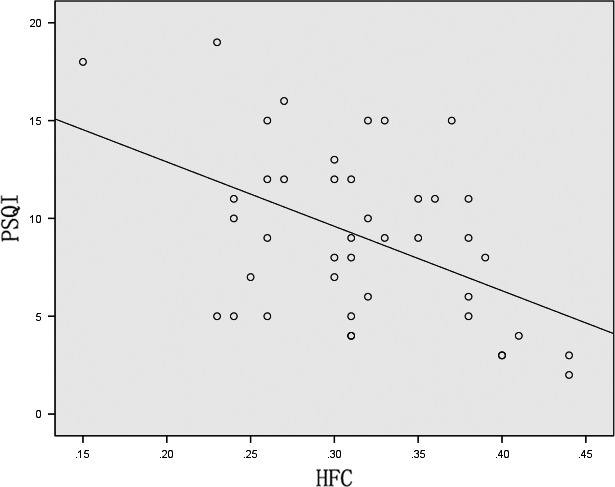
Correlation of the PSQI score and HFC ratio in PD group

The PSQI score showed a negative correlation with the HFC ratio (*r *= −.478, *p *= .001) (Figure 3) although no significant correlation was observed between the LFC and VLFC (*r *= .057, *p *= .719 and *r *= .193, *p *= .220, respectively) in the PD group.

### 3.5 Logistic regression analysis of PD group with poor sleep quality

Factors that may affect the occurrence of PD in patients presenting with poor sleep quality (18 PD patients) were included in the logistic regression analysis. Our results showed that the UPDRS score (odds ratio [OR] 1.241, 95% confidence interval [CI] 1.018–1.513, *p *< .05) and nocturia (OR 11.810, 95% CI 1.345–103.725, *p *< .05) were significantly associated with the occurrence of PD in patients with poor sleep quality.

## 4 DISCUSSION

The key findings of our analysis are as follows: (1) The PD group showed lower HFC and sleep efficiency but higher VLFC and sleep latency than that observed in the control group. (2) The PD group showed significantly higher PSQI scores than those observed in the control group. (3) A negative correlation was observed between the HFC ratio and subjective indicators of sleep quality. (4) The severity of PD and nocturia were observed to be independent risk factors for the occurrence of PD in patients with poor sleep quality.

Physiologically, sleep structure undergoes significant changes with aging including a reduction in the total nocturnal sleep time and REM sleep, delayed onset of sleep, and a low arousal threshold (Roepke & Ancoli‐Israel, 2010). As a neurodegenerative disorder, PD may show a high prevalence of these similar changes. Our study showed a poor overall sleep quality in patients with PD based on both, CPC analysis and PSQI scores. Moreover, CPC sleep quality variables were associated with subjective sleep quality and the severity of PD, particularly in terms of the stable sleep component. These findings may enhance the utility of this ECG‐based method to evaluate sleep disorders in PD patients.

Furthermore, our study showed that the severity of PD and the frequency of nocturia affect sleep quality in patients with PD. Rigidity and akinesia both contribute to inability/difficulty to turn in bed, which has been rated the most troublesome nocturnal symptom reported by 65% of the 220 patients with PD who were investigated in a previous study (Lees, Blackburn, & Campbell, 1988). Additionally, DA agonists and L‐dopa used to treat PD can cause sleep disorders in various ways (Fahn, Jankovic, & Hallett, 2007). A possible reason is that antiparkinson medicines do not work at midnight as the dose in the blood decreases and this leads to motor, sensory, psychiatric, or autonomic symptoms such as nocturia. Additionally, antiparkinson medicines can cause vivid dreams, hallucinations, and paranoia, particularly at night. In contrast to the above‐mentioned study, we did not observe any correlation between the DA dose and the sleep quality. A possible explanation for this finding could be that we did not use PSG to record muscle tone in patients with PD at night; therefore, we could not confirm the correlation between the DA dose and motor symptoms.

The mechanisms causing stable sleep reduction in patients with PD remain unclear. However, the pathophysiology of sleep disorders in PD may be explained as follows: It is well known that sleep is regulated by a complex interplay between several neurochemicals via genetic and molecular regulation (Moore, 1997). DA, serotonin, norepinephrine, acetylcholine, and neuropeptides such as hypocretin (Siegel, 2009) appear to be involved in the modulation of the sleep–wake cycle. DA and serotonin are known to promote waking and suppress slow‐wave and REM sleep (Monti & Jantos, 2008). Hypocretin‐producing neurons are widespread throughout the central nervous system including the septal nuclei, locus coeruleus, substantia nigra, and the thalamus, where they stimulate arousal systems including neuroendocrine, metabolic, and autonomic pathways (De & Sutcliffe, 2005). Thannickal et al. reported a reduced number of hypocretin‐producing neurons in the hypothalamus in patients with PD and that the severity of hypocretin loss correlated with the clinical stage of PD. The authors concluded that this pathomechanism could play a role in the causation of sleep disorders in PD (Thannickal, Lai, & Siegel, 2007). Their results were consistent with our findings in that the severity of disease was observed to be an independent risk factor for the occurrence of sleep disorders in patients with PD.

Conventionally, PSG has been widely used for sleep monitoring. However, quantified PSG variables may not effectively differentiate between normal sleepers and patients with sleep disorders (Rechtschaffen & Kales, 1968; American Psychiatric Association (APA), 1994), or the changes could be negligible and the correlations with overt clinical symptoms could be weak. CPC analysis, based on the Fourier principle evaluates the coherence between sleep and respiration by calculating the effects of HRV and the changes in the amplitude of the ECG R‐wave induced by alterations in tidal volume. This method deduces the coherence of sleep. CPC classifies sleep as stable and unstable, which is independent of the standard sleep phases. HFC occurred during NREM 2 and NREM 3 and respiratory stability and decreased with increasing sleep‐related episodes such as sleep apnea (Thomas et al., 2009), fibromyalgia, (Thomas et al., 2010) and depression (Yang et al., 2011). CPC analysis eliminates the need for arbitrary scoring by directly measuring biologic signals and provides a unique perspective for an understanding of sleep physiology and pathology. Moreover, previous data (Thomas et al., 2005) have shown a significant correlation between PSQI and CPC variables and CPC can quantitatively measure the sleep quality of subjects. From a research viewpoint, CPC analysis could objectively measure sleep quality in those with sleep disorders and be used to describe the longitudinal course of sleep disorders in clinical practice.

Limitations of our study are as follows: 1) PSG was not performed; thus, we could not determine an accurate correlation between conventional sleep indices. Differentiating between REM sleep and the wakeful state was difficult. 2) Our control group showed distinctly lower HFC values (36.37%) than expected normal values (≥ 50%) in adults, which could lead to statistical insignificance. 3) Our study included a small number of patients with PD as well as controls, which may not conclusively support our findings.

## 5 CONCLUSION

Our study reflects a poor overall sleep quality in patients with PD based on both, CPC analysis and PSQI scores. Additionally, we observed that CPC analysis reflects the subjective sleep quality of patients with PD. CPC analysis can serve as a novel sleep monitoring tool to screen patients with “good” and “poor” sleep quality.

## CONFLICTS OF INTEREST

None.
